# The Measurement of Polymer Swelling Processes by an Interferometric Method and Evaluation of Diffusion Coefficients

**DOI:** 10.3390/ijms11020532

**Published:** 2010-02-03

**Authors:** Aleš Mráček

**Affiliations:** Department of Physics and Material Engineering, Faculty of Technology, Tomas Bata University in Zlin, Nad Stráněmi 4511, 760 05 Zlín, Czech Republic; E-Mail: mracek@ft.utb.cz; Tel.: +4-206-087-075-77; Fax: +4-205-760-351-02

**Keywords:** swelling, interferometry, image analyses, concentration profile, diffusion, hyaluronan

## Abstract

The amorphous polymer film swelling in a liquid solvent below the glass transition temperature was characterized by a few kinetic parameters (especially the mutual diffusion coefficient of swelling and its mean value) obtained by interference of monochromatic light in the wedge arrangement. This interferometric method allows one to determine the concentration field in the swollen surface layer and consequently to compute the concentration-dependent diffusion coefficient. A software system developed at the Department of Physics and Material Engineering at TBU in Zlin has been used for the evaluation of the main kinetic parameters of the swelling process. The software can be used for the on-line analyses of interferograms during the swelling process. The main application outputs are the computation of the concentration profile, the concentration gradient, the mutual diffusion coefficient of the swelling by the solvent and its mean value.

## 1. Introduction

The optical interference methods associated with the measurement of free-diffusion are very old experimental techniques. During the sixties of the last century, several authors published papers describing a thin wedge interferometer [1–4]. This method is based on the measurement of the refractive index distribution and thanks to the wedge interferometer, can be used for the determination of the concentration or the concentration gradient distribution. The diffusion cell is an integral part of the wedge interferometer apparatus. The light interference arises in the semi-transparent cell walls which are arranged to form a thin wedge where the free diffusion proceeds. The wedge apparatus is applicable to studies of the diffusion process of polymer swelling by a solvent. Moreover, the concentration distributions can be measured over very small distances in the surface swollen layer (solid swollen layer [5,6]). Nevertheless, one significant limitation of the technique is that the absolute difference between the refractive index of the polymer (*n*_p_) and the refractive index of the solvent (*n*_s_), Δ*n* = *n*_p_ − *n*_s_, must be larger than 0.1.

Recently, papers describing a very similar interferometric method used on swelling of polymer films were published by Goossens, *et al.* and Manoli, *et al.* [7,8]. In this paper, the construction of interferometer, the theoretical background of the interferometric method and a user-friendly computer system for the evaluation of the obtained data describing the diffusion process of the swelling by an interferometric method are described.

### 1.1. Theoretical Background of the Experimental Technique

The phenomenon of light interference in thin layers (films) or the thin wedge layers is described by the principles of fundamental wave optics [9,10,12]. The interference of electromagnetic waves (in the described interferometer monochromatic light, *λ* = 589 nm, was used) reflected from both interfaces of layer and the number of the interference lines depends on the refractive index of the layer [9–11]. The refractive index of polymers in solutions depends strongly on their concentration [4,5,11,]. These are the fundamentals of the presented interferometric method, because the refractive index of the surface swollen layer is changed with the concentration of the polymer in this layer during the diffusion process of the swelling.

**Figure 1.**
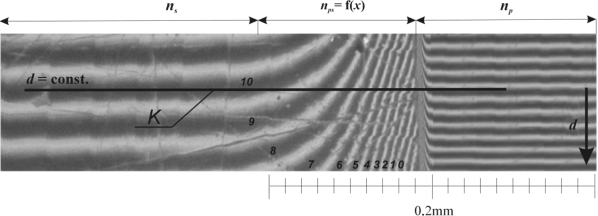
Interferogram obtained by the constant wedge width technique and its interpretation (sample of Hyaluronan at 37 °C in t = 210 seconds); ns – refraction index of pure solvent; np–refraction index of polymer; nps–refractive index in SSL; 0, 1, 2,…,K–number of crossed lines; d–width of wedge.

The interferometric method enables us to determine the shape of the concentration field in a swollen surface layer (SSL) [5,6] by two experimental procedures, *i.e*., the method of constant optical width (the measured polymer samples have a constant thickness in any position) or the constant width of wedge method (the thickness of the measured polymer samples is growing perpendicular to the interference lines). The constant width of wedge technique was applied in this study and the experimental arrangement of the interferometric device can be seen in Figure 5 (examples of the interferograms are shown in Figures 1 and 6). The benefit of this method is that it enables one to obtain the concentration at an arbitrary number of points for constructing the curve of the concentration distribution (the relation between the polymer concentration in SSL and the position in SSL):

(1) $$c_{ps}=c_{ps}(x)$$

where *x* is the coordinate of position and *c_ps_* is the mass fraction of measured polymer.

The free-diffusion process was first discussed by Boltzmann and Matano [6,14,15], and it is possible to find the functional dependence of the diffusion coefficient on the concentration from the concentration gradient obtained by an interferometric measurement. The mutual diffusion in a system polymer-solvent with a one-dimensional experimental arrangement is described by the second Fourier-Fick law (system with concentration dependent diffusion coefficient) in the form [21]:

(2) $$\frac{\partial c}{\partial t}=\frac{\partial }{\partial x}\left(D(c)\frac{\partial c}{\partial x}\right)$$

where *t* is time and *c* is the concentration of the polymer. The solution for the following initial and boundary conditions:

(3) $$\begin{array}{lll} c=c_{0} & x>0 & t=0\\ c=0 & x<0 & t=0\\ c=c_{0} & x\to \infty & t>0 \end{array}$$

arising from the Boltzmann solution [14] of partial differential Equation (2) and its mathematical application put forward by Matano [15]. Boltzmann has generalized the volume change effect. This generalization has shown that in some cases of the volume change can be quite significant [5,13,14,16,17]. The hypothesis of the free-diffusion conditions of a sharp initial boundary and one-dimensional unrestricted diffusion can be expressed, then the concentration in swollen surface layer is a function only of the variable *η* (the meaning - the square root of the diffusion coefficient, thus *D*^1/2^):

(4) $$\eta ={\left(x/2t\right)}^{1/2}$$

where *x* is distance from initial boundary at which contact occurs and *t* is the time of swelling. After *x-*coordinate system has been properly established from interferograms, the concentration versus *x* data for these times is combined to form a single concentration versus *η* curve [4]. By using Equations (2) and (4) is possible to derive a convenient form of generalized Equation for the analysis of the wedge data:

(5) $$D(\eta )=\frac{1}{\rho }\frac{\text{d}\eta }{\text{d}\phi_{1}}\left(-\int_{\rho_{10}}^{\rho_{1}}2\eta \text{d}\rho_{1}+\phi_{1}\int_{\rho_{0}}^{\rho }2\eta \text{d}\rho \right)$$

where *D*(*η*) is the mutual diffusion coefficient when the mass density of a species 1 is *ρ*_1_; *ρ* and *φ*_1_ are the total solution density and the mass fraction of a species 1 at this composition, respectively; and *ρ*_0_ and *ρ*_10_ are the total density and the species density, respectively, at the limit of the diffusion field where the solution velocity is zero[4]; and *η* is described in the Equation (4). In the study of Duda *et al.* [4]: “the closed end of graphite channel used for creation of constraining boundary which maintained a zero velocity at this limit of the free diffusion field” (there was used two boundary graphite wafers in experimental setup which determinate ends of sample, see in Figure 2 in [4]).

**Figure 2.**
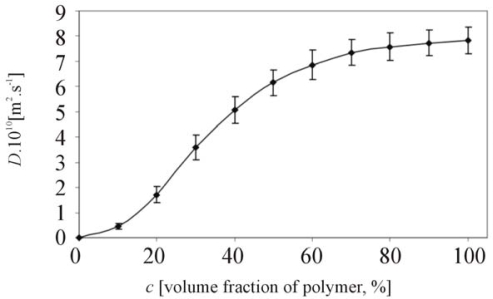
Dependence of mutual diffusion coefficient on concentration of polymer (the example of hyaluronan swelling at 37 °C).

**Figure 3.**
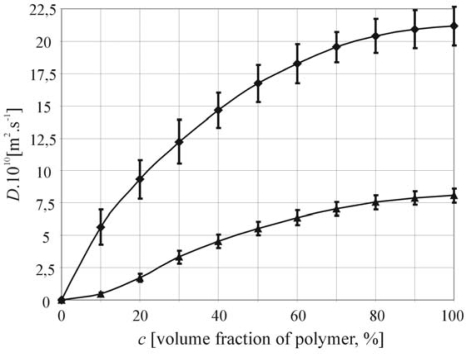
The Comparison of mutual diffusion coefficient dependent on concentration of hyaluronan for two molar masses (diamonds-M_w_ = 50.28 kDa and triangles-M_w_ = 1377 kDa).

The refractive index at any position *x* in the diffusion field [18,19] can be related to the refractive indices at two limits by an Equation derived by Paul [1]:

(6a) $$\frac{n(x)-n(\infty )}{n(-\infty )-n(\infty )}=\frac{1-\xi }{[n(-\infty )/n(\infty )-1]\xi +1}$$

where:

(6b) ξ=[y(x)−y(−∞)]/[y(∞)−y(−∞)]

where *y* is the perpendicular position on the position *x* in the image of an interferogram, can be seen in Figures 2 and 3) [1]. The Equations (6a, 6b) was derived for the evaluation of measuring along one fringe (the interference line) or path of constant optical thickness (the thickness of the measured polymer sample). The example of monochromatic light interference in the thin layer can be seen in the Figure 2 where the dark and light regions are the result of destructive and constructive interference [1,4,5,9,10,12]. Other researches [11,19–20] have explicated wedge fringe patterns by the zero value of position and the number (*K*) of crossing interference lines along a reference line (*d* = const., see in Figure 2) which is parallel to the axis of the wedge in the direction of diffusion. The evaluation principle of the refractive index as a function of position in the surface swollen layer is represented by Equation (7) and (8). The straight line coalescing with parallel sections in pure polymer and solvent is drawn through the diffusion field obtained. The necessary condition of monochromatic light interference is the coherent source of light and the interference fringes (light lines in Figure 2) arise if the following Equation is fulfilled as:

(7) $$m\lambda =2d(n_{p}-n_{s})$$

where *m*, *λ*, *d*, *n_p_* and *n_s_* are an integer, the wavelength, the width of the wedge, the refractive index of the pure solvent, and the refractive index of the polymer, respectively. The coalescence of parallel reference lines (*d* = const., see in Figure 2) in polymer with interference fringes in solvent is caused by perfect optical isotropy of particular regions of the swollen surface layer. If *K* is the number of lines (see Figure 1), which intersect the reference line (*d* = const.), then the following Equation is valid for the refractive index in the *i*-th point of intersection in *ps*-region (the surface swollen layer):

(8) $$n_{ps,i}=n_{p}+i\frac{n_{s}-n_{p}}{K}$$

By measuring the position of the *i*-points of intersection in relative scales (with respect magnification) and using Equation (8), the curve *n = n*(*x*) may be constructed. The accuracy of the *n*-*c* transformation (refractive index to concentration) depends on the number of lines intersecting the reference line [number of lines = f(*n_p_* – *n_s_*)] and the distance between the semi-transparent glass plates. The wedge microinterferometer may be used only if the refractive index of solvent *n_s_* is different from refractive index of polymer *n_p_*. In this case, two spots with different concentrations have different indices of refraction. The change in refractive index is proportional to the concentration change in a not too wide concentration interval. The preliminary interferometer data can easily be converted to give the form of the concentration distribution (the refractive index as a function of concentration) at each time that an interferogram is obtained. On the other hands, several author determined refraction index-concentration relation more precisely [22,23]. Their experimental setup of the measurement apparatus enables direct determination of refractive index-concentration relation. The n-c relations go through a maximum in low concentration region, in line with what can be expected when excess free volume was being filled, as their results show [22,23].

The mean value of the mutual diffusion coefficient *D*_mean_ is more simple for computation and the formula for this variable can be written as [11]:

(9) $$D_{\text{mean}}=x^{2}/2t$$

where *x* is the surface swollen layer (SSL) and *t* is time when SSL was measured.

## 2. Results and Discussion

**Figure 4.**
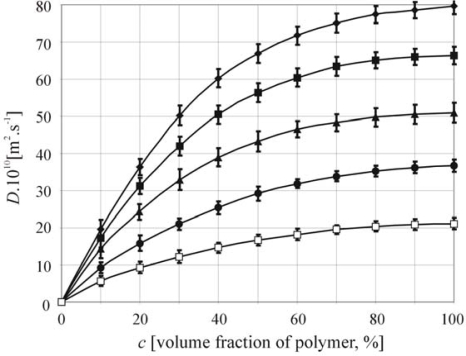
The Comparison of mutual diffusion coefficient dependent on concentration of hyaluronan (M_w_ = 50,28kDa) for five temperatures (diamonds-T = 45 °C, square-T = 40 °C, triangles-T = 35 °C, circles-T = 30 °C and white square-T = 25 °C).

In general, the developed interferometer and system for the analysis of interferograms is a suitable method to analyse the primary data image collections with the following evaluation of kinetics and static parameters of the swelling process for various systems polymer-solvent. The examples of *D* = *f*(*c*) can be seen in Figures 2–4. The sample of hyaluronan about M_w2_ = 850 kDa (the same sample in Figure 6) was swelled by water at 37 °C (Figure 2). The same system of hyaluronan-water (Figure 2) was used in [11]. In Figure 2, the values of mutual diffusion coefficient are dependent on volume fraction of polymer. However, the data published in [11] are mean values of mutual diffusion coefficients calculated by Equation 9.

The comparison of two molar mass of hyaluronan (M_w1_ = 50.28 kDa and M_w2_ = 1,377 kDa) swelled by water at 25 °C can be seen in the Figure 3.

Finally, the comparison of hyaluronan (M_w2_ = 50,28 kDa) swelled by water at five temperatures (25 °C, 30 °C, 35 °C, 40 °C and 45 °C) is illustrated in Figure 4. All error bars estimated for confidence interval at the significant level 95% stand for the standard errors for the nine different measurements of the same sample. These results (Figures 2–4) are corresponding to the theory of the swelling diffusion process and published data [5,11,13,16,17,24]. Wik and Comper [17] published very similar data for the concentration dependence of the diffusion coefficient in system hyaluronan-water in dilute and semi-dilute solutions. The mutual diffusion coefficient measured by Wik and Comper was analyzed by boundary relaxation in analytical ultracentrifuge by either Raleigh interference optics and absorption optics through the use of fluorescein-labeled hyaluronate. Their experimental data partly confirmed that the mutual diffusion coefficient increased with increasing polymer fraction. Our software system enables to keep count of whole polymer concentration range.

Mean mutual diffusion coefficients are shown at the Tables 1 and 2.

These values support the widely known fact, that the diffusion coefficient increases with increasing temperature (Table 1) or decreasing molar mass (Table 2) [11,21].

## 3. Experimental Section

### 3.1. Materials

All materials and chemicals used for the measurement were of the standard purity p.a. Three samples of Sodium hyaluronate (M_w1_ = 50.28 kDa, M_w2_ = 850 kDa, M_w3_ = 1377 kDa) was obtained from the Contipro, Ltd., Czech Republic. The diffusion process of swelling was studied on the solid films of the polymer samples prepared by casting from the 0,01M concentration solutions in redistilled water and by the subsequent evaporation in a dessicators. The redistilled water was used as the solvent for the measuring of swelling kinetics.

### 3.2. Experimental Arrangement and Measuring

**Figure 5.**
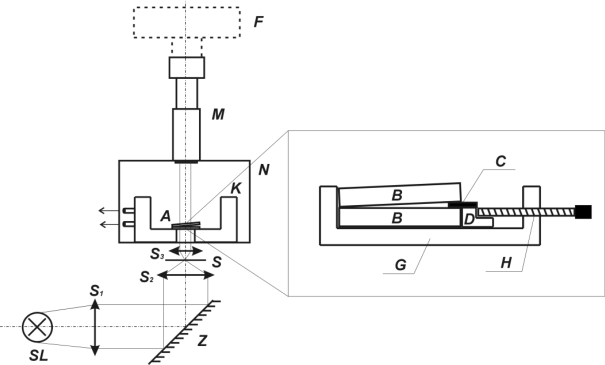
Schema of interferometer. SL – sodium-vapour lamp, S1–S3 – lenses; Z – mirror; S – slit; K – temperature-controlled vessel; N – base of microscope; M – microscope; F – CCD camera; A – holder of glass plates with sample; B – optically flat (coated glass plates, between this glasses was put measured sample); C – wedge spacer which determines the wedge angle; D – positioning block which slides horizontally in a dovetail channel; G – cell which is positioned on microscope stage; H – screw for adjustment of wedge angle.

The experimental arrangement of the interferometer and the cell, which is positioned on the microscope stage, can be seen in Figure 5. The films were prepared from the Hyaluronan by pouring its solution in the redistilled water in Petri dish. This film was dried in the vacuum for 24 h at 25 °C and subsequently held in the desiccator. The analytical grade distilled water was applied as the solvent. The samples were cut off these films and as thin plates of rectangular cross-section having the area of 2.10^−5^m^2^ and the width of 8.10^−5^–6.10^−4^m. They were put in a the temperature-controlled holder of the volume of 1,6.10^−6^m^3^ between two semi-transparent glass plates with the transparence of about 40%. The sample was so pressed by means of torsion springs so that the density of lines (the internal pressure) was the same for each measurement. The time was always measured from the moment when the thermostatted solvent was injected. The temperature was held constant with deviation ±0.1 °C. In each measurement, 3–5 shots were taken at convenient time intervals. The resulting interferograms were scanned by the digital camera NIKON COOLPIX 4500.

**Figure 6.**
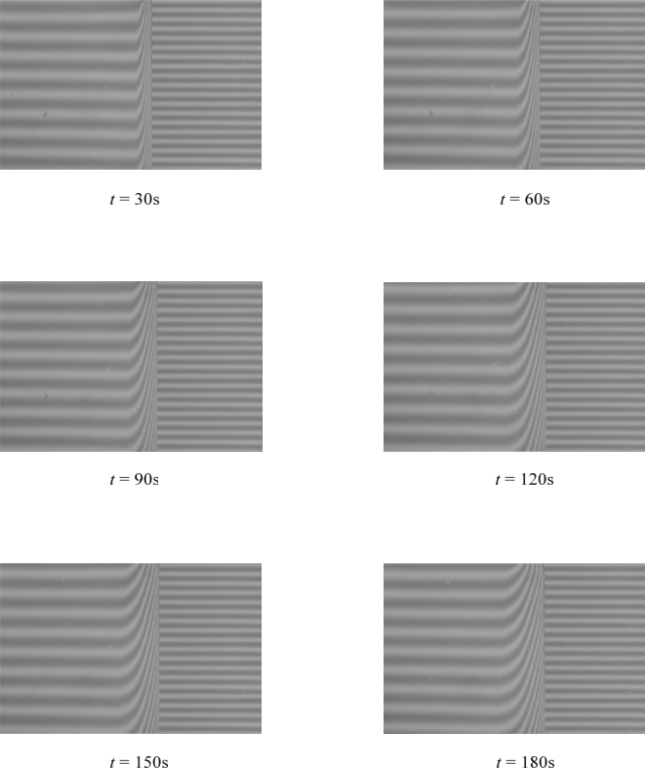
Swelling process illustrated by interferograms in time interval 30–180s.

The observed diffusion process of the solvent into the polymeric material is recorded in the form of the interferograms using appropriate equipment of the experimental framework and stored in the image files (see Figure 6).

### 3.3. Interferogram Analyses

The computer system called SAIA 3.0 (System for Analysis of Interferograms) for analysing of obtained interferograms was developed at Department of Physics and Material Engineering, TBU in Zlin. Therefore, the data describing the diffusion and the swelling process could be evaluated by the computer software. The SAIA 3.0 is based on the simple theoretical background of the interferometric method and has been created in a graphical development enviroment NI LabVIEW 7.1. The functions of image analyses have been realized by NI Vision tools. LabVIEW is widely expanded and used in the industrial and the scientific area [25–29]. The final version of the system has been converted to a standard Windows application and supports standard image file formats (APD, BMP, JPEG, PNG and TIFF) as data input. An output of analyzed data can be saved via frequently used ASCII text code or spreadsheet format, modified image can be saved in the same formats as it was loaded. The support files with temporary data sets use XML format. The interferograms acquired by the digital camera associated with the wedge interferometer are analysed to calculate the refractive index distribution along flow coordinate axis and then a distribution of the concentration. The main idea of this part is to identify the flowline between a polymer and the solvent area by recording coordinates (see Figure 2). The obtained data is consequently submitted to the statistical computations, especially the regression analysis. From aquired camera images, the SAIA software is able to determinate the refractive indexes and the essential static and the kinetic parameters of the polymer swelling.

## 4. Conclusion

This paper presents very useful and cheap experimental method for determination concentration gradient in the diffusion process of the polymer swelling by the thermodynamically compatible solvent. There were observed sharp increase of diffusion coefficient with polymer concentration. The interferometric method was described by several authors [4,7,11]. Nevertheless, the evaluations of the kinetic variables from interferograms obtained by this method are very time-consuming. The wedge interferometric method with software SAIA 3.0 (developed by A. Mracek and P. Urban–Department of Physics and Material Engineering, Faculty of Technology, Tomas Bata University in Zlin, Czech Republic) [11] becomes a new and progressive exercise in scientific work.

## Figures and Tables

**Table 1.** The mean mutual diffusion coefficients (D_mean_) of hyaluronan (M_w_ = 50.28 kDa) for five different temperatures. (The standard errors estimated for confidence interval at the significant level 95% was computed for 5 measurements of the same sample).

**T [°C]**	***D*_mean_ [m^2^.s^−1^]**	**S.E.**
25	1.6 × 10^−9^	5 × 10^−10^
30	2.7 × 10^−9^	8 × 10^−10^
35	4 × 10^−9^	9 × 10^−10^
40	5.2 × 10^−9^	7 × 10^−10^
45	6.2 × 10^−9^	7 × 10^−10^

**Table 2.** The comparison of the mean mutual diffusion coefficients (Dmean) of hyaluronan for two molar mass. (The standard errors estimated for confidence interval at the level 95% was computed for 5 measurements of the same sample).

***M*_w_ [kDa]**	***D*_mean_ [m^2^.s^−1^]**	**S.E.**
50.28	1.62 × 10^−9^	5.0 × 10^−10^
1,377	5.23 × 10^−10^	4.4 × 10^−11^
